# Methods for Observed-Cluster Inference When Cluster Size Is Informative: A Review and Clarifications

**DOI:** 10.1111/biom.12151

**Published:** 2014-01-30

**Authors:** Shaun R Seaman, Menelaos Pavlou, Andrew J Copas

**Affiliations:** 1 MRC Biostatistics UnitCambridge CB2 0SR, UK; 2 Department of Statistical Science, University College LondonLondon WC1E 6BT, UK; 3 MRC Clinical Trials Unit at University College LondonLondon WC2B 6NH, UK

**Keywords:** Bridge distribution, Immortal cohort inference, Informative missingness, Missing not at random, Mortal cohort inference, Semi-continuous data

## Abstract

Clustered data commonly arise in epidemiology. We assume each cluster member has an outcome *Y* and covariates 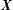. When there are missing data in *Y*, the distribution of *Y* given 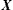 in all cluster members (“complete clusters”) may be different from the distribution just in members with observed *Y* (“observed clusters”). Often the former is of interest, but when data are missing because in a fundamental sense *Y* does not exist (e.g., quality of life for a person who has died), the latter may be more meaningful (quality of life conditional on being alive). Weighted and doubly weighted generalized estimating equations and shared random-effects models have been proposed for observed-cluster inference when cluster size is informative, that is, the distribution of *Y* given 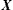 in observed clusters depends on observed cluster size. We show these methods can be seen as actually giving inference for complete clusters and may not also give observed-cluster inference. This is true even if observed clusters are complete in themselves rather than being the observed part of larger complete clusters: here methods may describe imaginary complete clusters rather than the observed clusters. We show under which conditions shared random-effects models proposed for observed-cluster inference do actually describe members with observed *Y*. A psoriatic arthritis dataset is used to illustrate the danger of misinterpreting estimates from shared random-effects models.

## 1. Introduction

Clustered data are common in epidemiology. Repeated measures are clustered in individuals; teeth in patients; pups in litters. Suppose interest is in the association between outcome *Y* and covariates 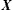 measured on members of the clusters. Often *Y* and 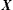 are missing for some members of sampled clusters. For simplicity, we assume that a member's 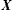 is observed whenever *Y* is observed. We call members with observed *Y* “observed members,” those with missing *Y* “missing members,” the original clusters “complete clusters,” and the subclusters that remain after discarding missing members “observed clusters.”

Missing data may arise because although a variable could, in principle, be measured, circumstances meant it was not, for example, because an individual missed a visit. We call such missing data “potentially observable.” When missing data are potentially observable, a model can be proposed for the distribution of *Y* given 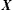 in all cluster members, and methods used that, under specified assumptions about the missingness (e.g., missing at random, MAR), give consistent estimates for this model. We call this “complete-cluster inference.”

Alternatively, missing data may arise because in a fundamental sense a variable does not exist. We call such missing data “unobservable.” Three examples of unobservable *Y* are measures of: (1) cognitive function of an individual after death; (2) degree of disablement of an individual who is not disabled; (3) health of a tooth that has been lost. Although missing *Y* could be set to zero when a patient is dead/not disabled/tooth is lost, in practice often a model is instead proposed for *Y* given 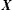 in observed members only (so conditional on alive/disabled/tooth not lost). We call this “observed-cluster inference.” Sometimes observed-cluster inference may be of interest even when missing data are potentially observable. When missing data are unobservable “complete-cluster” inference is philosophically problematic: what does it mean to model cognitive function in dead people?

When the size *M* of complete clusters varies, it is usually assumed that *Y* is independent of *M* given 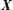. In observed clusters, however, *Y* and *N* may be conditionally dependent given 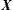, where *N* is size of observed cluster. For example, in a dental study, the fewer teeth a patient has, the worst their condition tends to be. This is called “informative cluster size” (ICS).

So far we have assumed observed clusters are generated from complete clusters by excluding missing members, but ICS can also arise where observed clusters are complete in themselves. For example, in toxicology, exposed dams who are more sensitive to a toxin may tend to have smaller litters and offspring with greater probability of deformation than less sensitive dams, so that *Y* (pup being deformed) and *N* (litter size) are dependent given *X* (exposure of dam).

We shall show that three of the methods proposed for observed-cluster inference under ICS, viz. weighted and doubly weighted generalized estimating equations (GEE) and shared random effects models, can be seen as actually giving inference for complete clusters. When the *Y*’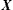 associations in complete and observed clusters are the same, the distinction is unimportant. However, ICS causes them to differ in general. So, it is important to understand when methods proposed for observed-cluster inference really do describe observed clusters. In the literature on modeling repeated measures in cohorts with high death rates (Dufouil et al., 2004; Kurland et al., 2009) a distinction has been made between complete-cluster (termed “immortal-cohort”) inference and observed-cluster (“mortal-cohort”) inference. However, conditions under which the two inferences are equivalent have not been set out, and in the wider literature the distinction seems to be less well recognized.

In Section 2011 we define notation and discuss methods for complete-cluster inference from observed data. Section 2004 defines ICS and discusses how ICS relates to missing-data mechanisms. Section 2011 relates two weighted GEE methods, one proposed for complete-cluster inference in the missing-data literature, and one for observed-cluster inference in the ICS literature. We also show that doubly weighted GEE, proposed for observed-cluster inference, actually give complete- rather than observed-cluster inference, and that, moreover, there is no single complete-cluster inference. Shared random-effects models give complete-cluster inference, but have also been used for observed-cluster inference. In Section 2011 we discuss when this is valid, and in Section 2011 we use a psoriatic arthritis dataset to illustrate that some parameters of such a model may be relevant to observed clusters but others not. In brief, we replicate an analysis of association between disability and covariates, with measurements clustered by patient. Our interest is in how sex affects degree of disability in the “observed clusters” of measurements where degree is greater than zero, that is, given disability. The analysis uses models for probability of disability and for degree of disability given disability which share a random intercept. Because probability of disability is higher in women than in men with the same intercept and other covariates, intercept and sex are not independent given disability and other covariates. Consequently, the effect of sex on degree of disability given disability is less than is suggested by the estimated parameter.

## 2. Notation and Complete-Cluster Inference

Let *K* be the number of complete clusters in the sample. When needed we use subscript *i* to index cluster, but usually omit this. Let *M* (known) be size of complete cluster. Let 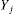 and 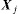 (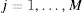) be outcome and covariate vector, respectively, for member *j* of the complete cluster, and 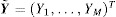 and 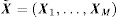. Let 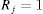 if 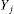 is observed, 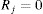 if 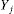 is missing, and 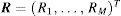. 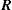 is always observed. Members with 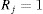 are “observed members”; those with 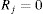 are “missing members.” Let 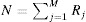 be size of observed cluster. Assume 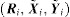 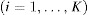 are i.i.d. For any value 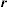 of 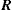, partition 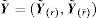, where 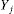 belongs to 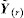 if 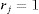 and to 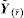 if 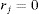. For example, 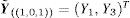 and 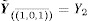. Partition 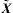 likewise, except that if some elements of 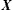 are observed even on missing members, these elements belong to 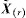.

Data are missing at random (MAR) if 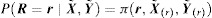 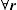 for some function 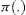 (informally, 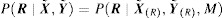) and missing completely at random (MCAR) if 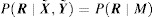 (Seaman et al., 2013) (note *M* is a function of 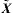, as 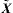 has *M* columns). Otherwise they are missing not at random (MNAR). We say data are missing with equal probability (MWEP) if 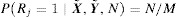 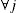. MCAR means that which members are observed does not depend on 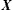 or *Y* values in the cluster. This would be so if, for example, missing data had been lost by the researchers. MAR allows missingness to depend on data on observed members plus any observed data on missing members. For example in a longitudinal study individuals’ probability of dropout may depend on past health measurements but not on current health. If it also depends on current health, the data are MNAR. MWEP means the number *N* of observed members may depend on 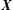 and *Y* but given this number all sets of *N* observed members are equally likely. This could be so if missingness depends only on cluster-level summaries of 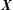 and *Y*.

The missingness process is monotone if 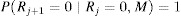 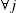. 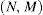 then defines 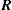 and *vice versa*. If 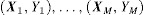 are exchangeable given *M*, we say “members of complete clusters are exchangeable.” Indices 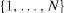 can then be assigned to observed members and 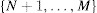 to missing members. Missingness is then monotone.

To make “complete-cluster” inference, a model is specified for 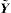 given 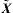. To fit this using observed data (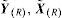), an assumption (e.g., MAR) is made about the missingness process and a method used that is valid under this assumption, for example, inverse probability weighting (IPW) or random-effect models (Albert and Follmann, 2009). We consider two approaches to complete-cluster inference that relate to methods proposed for observed-cluster inference. The first specifies a (marginal) model for 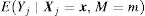 and assumes


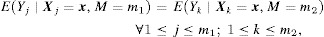
(1)

so that we can define 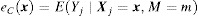. This model is fitted to observed clusters using GEE with IPW. The second approach uses a shared random-effects model. This gives cluster-specific inference, but random effects can be integrated out to get 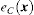.

## 3. Informative Cluster Size

### 3.1. Semi-Parametric Marginal Models

For each cluster with 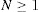, let *H* be the index of a randomly selected member of the observed cluster. So, 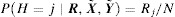. Marginal inference for the *population of typical observed members* and marginal inference for the *population of all observed members* mean estimating the parameters of a model for 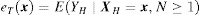 and for 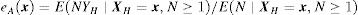, respectively. Whereas 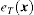 is the expectation of *Y* given 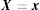 giving equal weight to each observed cluster, 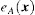 gives equal weight to each observed member. Clusters with 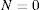 play no role in 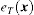 or 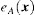.

Hoffman et al. (2001), Williamson et al. (2003) and Benhin et al. (2005) define non-informative cluster size (NICS) as 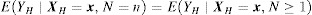 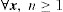. Otherwise cluster size is informative (ICS). Under NICS, 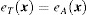 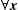. Under ICS, 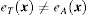 in general. They advocate using 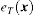. Use of 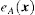 has been proposed for mortal cohorts when missing data are due to death, and for modeling degree of disability or health of teeth when missing data are due to non-disabled patients or absent teeth (Dufouil et al., 2004; Kurland et al., 2009; Su et al., 2011; Li et al., 2011). Hoffman et al. (2001) gave an estimator for 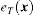. Williamson et al. (2003) and Benhin et al. (2005) gave an asymptotically equivalent and computationally less intensive method: weighted independence estimating equations (WIEE) (see also Wang et al. (2011) for three-level data). The same equations without weighting (IEE) estimate 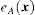. We describe WIEE and IEE in Section 2007.

### 3.2. Random-Effects Models

Dunson et al. (2003), Gueorguieva (2005), Chen et al. (2011), and Neuhaus and McCulloch (2011) consider cluster-specific inference using a linear or generalized linear mixed model (LMM/GLMM). They interpret NICS to mean the random effects 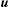 in the mixed model are independent of *N*, and ICS to mean they are not. NICS in this sense implies NICS in the sense of Hoffman et al., but the converse is not true. To deal with ICS when fitting the LMM/GLMM, several authors have combined it with a model for *N* or 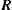, with the same or correlated random effect (Dunson et al., 2003; Gueorguieva, 2005; Chen et al., 2011; Su et al., 2009; Su et al., 2011; Li et al., 2011). We discuss this model in Section 2011.

### 3.3. Relating ICS to Missingness Mechanisms

Hoffman et al. (2001) wrote that ICS is “closely related” to violation of the MCAR condition. In fact, MCAR is not a sufficient condition for NICS. For example, suppose all complete clusters have size 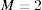 and have 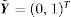, there are no covariates, and 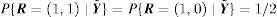. It is easy to show that 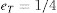 but 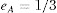.

**Proposition 1**

Cluster size will be non-informative if data are MCAR and, moreover, either i) equation 2009 holds, or ii) 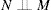 and the data are MWEP.

Note 2009 is often assumed with GEEs, but 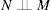 is unlikely, as 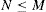. Proofs of Propositions are in Web Appendices A and E. Just as both ICS and NICS can arise from MCAR mechanisms, so they can from MAR and MNAR (examples in Web Appendix B).

When 2009 holds, so 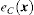 is defined, a sufficient condition for 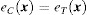 is MWEP and 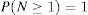, because the *Y*-*X* relation in a randomly chosen member of an observed cluster is then the same as in a random member of the corresponding complete cluster.

## 4. Weighted and Doubly Weighted GEE

### 4.1. Weighted GEE (WGEE)

Assume 2009 holds and 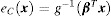, where *g* is a link function. If 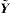 and 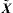 were observed, 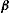 could be estimated with GEE. With missing data, WGEE can be used. These weight member *j* by 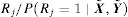. Robins et al. (1995) proposed use of WGEE when *M* does not vary, missingness is monotone and MAR, and 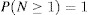.

When data are MWEP and 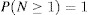, weights 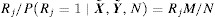 can be used instead (proof in Web Appendix C). In this case, 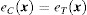 (Section 2001), so WGEE with weights 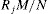 also give observed-cluster inference. In fact, with independence working correlation they are the WIEE proposed by Williamson et al. (2003) for estimating 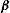 in 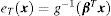. So, WIEE have a dual interpretation: they estimate 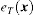 under any missingness mechanism; and 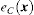 when data are MWEP and 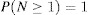.

WIEE without weights (IEE) estimate 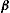 in a model 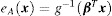 (Dufouil et al., 2004).

### 4.2. Doubly Weighted GEE (DWGEE)

If there is ICS and the distribution of 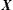 depends on *N*, interpretation of 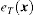 may be awkward, because the *Y*’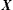 association is confounded by *N* (Williamson et al., 2003). For example, let *X* be binary and 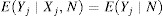 and 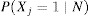 be increasing functions of *N*. Then typical members with 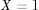 tend to come from larger clusters than typical members with 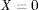, so 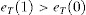 even though *X* has no effect on *Y* within clusters.

Huang and Leroux (2011) proposed DWGEE1 and DWGEE2. DWGEE1 can be used when 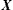 is categorical and every observed cluster contains at least one member with each of the possible values of 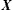. DWGEE1 are the same as WIEE except that member *j* is inversely weighted not by 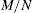 but by the total number of observed members in the same cluster who have 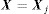. Thus the total weight of members with 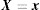 is the same for all possible 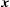. Rather than estimating 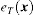, DWGEE1 estimate 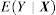 in the population formed by each cluster in the population contributing one member with each possible value of 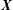.

DWGEE2 was proposed for when not all observed clusters contain a member with each possible value of 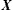. In DWGEE2 observed member *j* is inversely weighted by the *expected* (rather than *actual*, as in DWGEE1) number of observed members with 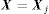. In Web Appendix D we show that DWGEE2 estimates 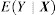 in a population of larger “complete” clusters in which each cluster contains at least one member with each possible value of 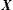. Each cluster in the dataset is considered to be the observed component of one of these larger clusters, with the rest being missing. The problem with this is that, unless observed clusters really do arise from larger clusters in which all values of 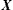 are represented (which is not so in Huang and Leroux's example), the larger clusters are purely hypothetical and it is unclear why they should be of scientific interest. Further, as shown in Web Appendix D, the distribution of *Y* given 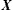 in the hypothetical population of complete clusters depends on which predictors are included in the model for the expected number with 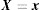, and there is no obvious reason to prefer one set of predictors to any other.

## 5. Random-Effect Models

### 5.1. LMM, GLMM, and Shared Random Effect Model

The general form of the LMM is (continuing to omit the subscript *i* for cluster)


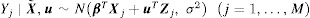
(2)


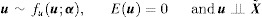
(3)


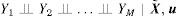
(4)

where 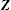 is a subvector of 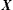, and 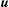 a cluster-specific latent variable. This is a model for *Y*’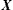 association in complete clusters. Assumption 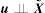 means that 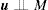 and hence that size of *complete* clusters is non-informative. Elements of 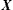 not in 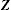 are said to have fixed effects; those in 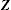 have random effects. It follows from 2005 and 2011 that 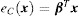. So, 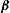 also has a marginal interpretation in complete clusters. LMMs are a special case of GLMMs. In GLMMs, 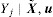 is assumed to belong to the exponential family, 2005 is replaced by


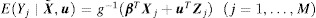
(5)

where 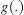 is the link function, and 2011 and 2004 are assumed to hold.

If *Y* is binary, 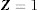 and 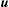 has a bridge distribution with rescaling parameter 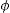 (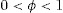), then 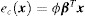 and so 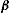 (in combination with 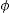) has a marginal interpretation in complete clusters (Wang and Louis, 2003). More generally, 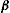 does not have a marginal interpretation, though 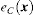 can be calculated as 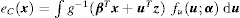.

The MLE of 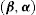 from fitting the mixed model to observed clusters is consistent when data are MAR, but not, in general, when MNAR. However, Neuhaus and McCulloch (2011) showed that for LMMs, if (i) 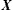 includes an intercept term, (ii) 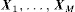 are i.i.d., (iii) 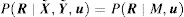, and (iv) the only random effect is an intercept (i.e., 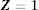), then 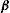 is consistently estimated except for the intercept. They found the same was approximately true of GLMMs. More generally, they say that if 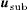 and 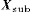 are subvectors of 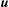 and 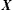 with 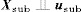 and 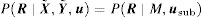, then their results suggest that the MLE of elements of 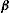 corresponding to 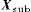 will be approximately unbiased.

For MNAR data, a model for 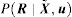 can be added to the LMM/GLMM. The result is a shared random-effects model (Albert and Follmann, 2009). When


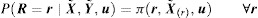
(6)

for some function 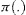, the MLEs of 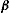 and 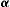 from this model are consistent. An indirect way (Su et al., 2009; Li et al., 2011; Su et al., 2011) to model 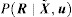 is to introduce another random effect 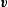, assume 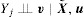, and specify models 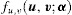 for the distribution of 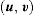 and 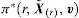 for 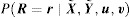. We call the resulting model for 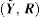 “a correlated random-effects model.” It is a special case of the shared random-effects model, with 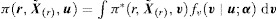 and 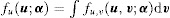.

### 5.2. Interpretation of 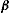 and 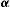 in Complete Clusters

Partition 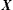 and 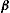 as 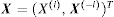 and 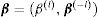, where 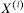 and 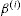 are the *l*th elements of 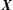 and 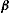, respectively. If 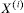 has a random effect, partition 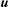 as 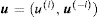, where 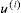 corresponds to 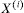, and partition 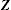 similarly. If 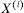 has a fixed effect, 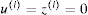, 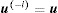 and 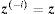. Let 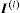 denote a vector of the same length as 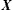, with *l*th element equal to one and all other elements equal to zero.

**within-cluster effects**

If 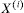 is cluster varying with fixed effect, 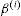 is its within-complete-cluster effect in clusters of size 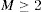. That is, if two members of the same complete cluster have 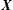 values that differ only by 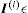 for some 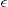, then their expected *Y* values differ by 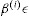 for an LMM. In a GLMM, the expected value is transformed by link function *g*; for example, for logit link, 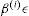 is their log odds ratio. If 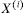 is cluster varying with random effect, 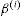 and 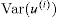 are the mean and variance of the within-cluster effect.

**between-cluster effects**


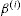
 and 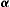 can be interpreted in terms of differences between expected *Y* in members of different complete clusters. That is, if for some 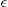, two complete clusters are randomly sampled conditional on one containing a member with 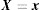 and the other a member with 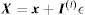, then the difference between the expected *Y* values of these two members is


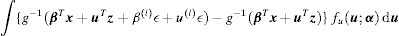
(7)

This reduces to 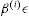 for the LMM and to 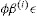 for the GLMM with bridge distribution.

**causal effects**

If 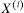 is manipulable, for example, treatment, 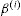 may be interpretable as a causal effect in complete clusters. Let 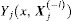 be the potential outcome of member *j* when 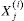 is manipulated to equal *x*. We make the following “causal assumptions” (Vansteelandt, 2007). First, 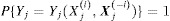, that is, observed outcome equals outcome that would be seen if 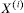 were set to its observed value. Second, manipulating 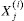 does not affect 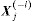 or 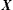 or *Y* values of other members. Third, 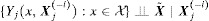, where 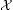 is set of possible values of 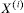. With these assumptions, the conditional expected causal effect 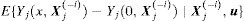 of 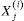 given 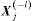 and 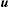 is 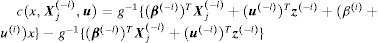. For LMMs, 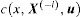 reduces to 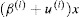. The conditional expected causal effect 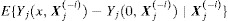 of 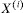 given 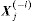 is 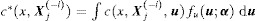, which reduces to 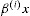 for LMMs and to 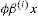 for GLMMs with bridge distribution.

### 5.3. Interpretation of 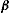 and 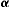 in Observed Clusters

Section 1995 discussed how 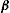 and 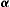 in the model defined by 2005–2004 or 2011–2003 describe the *Y*’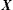 association in *complete* clusters. Now we discuss how the *same* 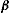 and 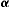 relate to associations in *observed* clusters.

**within-cluster fixed effects**

When 2005 holds and 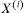 is cluster varying with fixed effect, 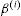 is not only the within-complete-cluster effect of 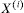, it is also the within-observed-cluster effect, which is the same in all observed clusters of size 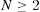. That is, if two members of the same *observed* cluster of size 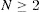 have 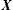 values that differ only by 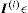 for some 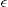, then their expected values (transformed by link function *g* in the case of the GLMM) of *Y* differ by 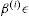.

When considering within-observed-cluster effects of covariates with random effects, between-observed-cluster effects and causal effects, we find it convenient to introduce the concept of the LMM/GLMM given by equations 2005–2004 or 2011–2003 “describing observed random subclusters.” For a cluster with 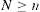, let 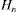 denote the set of indices of a simple random sample of size *n* from the *N* observed members, and let 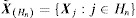. Note that 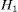 is the same as what we denoted in Section 2004 by *H*. We say “the LMM given by 2005–2004 describes observed random subclusters of size *n* from observed clusters of size 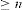
” (or, more concisely, “the LMM describes observed random subclusters of size *n*”) if


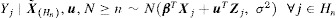
(8)


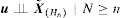
(9)


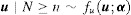
(10)


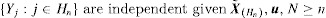
(11)

where 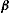 and 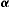 in 2011–2011 are the *same* parameters (i.e., have the same values) as in equations 2005–2004. Similarly, “the GLMM (given by 2011–2003) describes observed random subclusters of size *n*” if


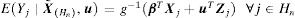
(12)

and 2007–2011 hold. If 2011–2011 or 2007–2011 hold for one or more values of *n*, we have a basis for interpreting the estimates of 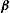 and 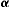 obtained by fitting the LMM/GLMM given by 2005–2003 (which describes *complete* clusters) in terms of effects in *observed* clusters. We give these interpretations below. Later (Proposition 2) we give sufficient conditions for the LMM/GLMM to describe observed random subclusters of size *n* and (Section 2008) show what can happen when these conditions are not satisfied. Note that the statement that LMM/GLMM describes random subclusters of size *n* is a statement about the *Y*’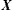 relation only in observed members of clusters with 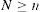; the association in missing members or in clusters with 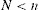 is not relevant. We shall focus on 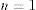 when discussing between-cluster effects, but for within-cluster effects we need 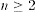, because within-cluster comparisons only make sense in clusters with at least two members. In most realistic settings, if the sufficient conditions (Proposition 2) are satisfied for *n*, they are also satisfied for 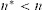.

**within-cluster random effects**

If the LMM/GLMM describes observed random subclusters of size *n* (with 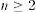) and 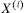 is a cluster-varying covariate with random effect, then 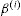 and 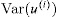 are the mean and variance of the within-observed-cluster effect of 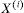. That is, if an observed cluster is randomly sampled conditional on 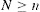 and on *n* members randomly chosen from it having 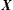 values that differ only in 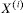, then the expected values (transformed by link function *g*) of *Y* of any pair of these *n* members differ by 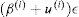, where 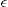 is the difference between their 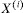 values, and the distribution of 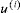 is given by 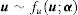.

**between-cluster effects**

If the LMM/GLMM describes observed random subclusters of size 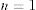, 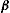 are the between-observed-cluster effects of 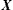. That is, if two clusters each with 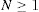 are randomly sampled conditional on 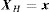 in one cluster and 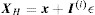 in the other, then the difference between the expectations of 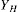 in the two clusters is


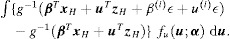
(13)

Since 1995 has the same form as 2001, between-cluster effects in observed and complete clusters are equal and 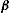 and 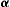 describe them both. As with 2001, 1995 reduces to 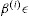 for the LMM. When 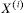 has fixed effect, this is true even if 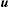 is not independent of *N*, so 2009 is not necessary for 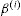 to be interpreted as a between-observed-cluster fixed effect in a LMM.

**causal effects**

Let 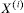 be manipulable and the “causal assumptions” of Section 1995 hold. Let 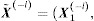 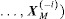 and 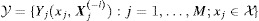. If the LMM/GLMM describes observed random subclusters of size *n* (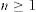) and 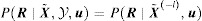, then 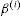 and 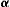 describe a causal effect of 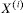 in observed random subclusters of size *n*. That is, the expected causal effect given 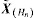 and 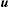 in the members whose indices belong to 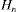 is equal to 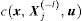 with 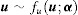, and the expected causal effect given 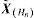 is equal to 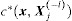. For the LMM when 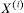 has fixed effect, 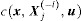 reduces to 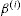 even if 2009 does not hold. Note that if 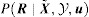 depends on 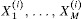, this causal interpretation is problematic because membership of observed clusters may change as 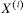 is manipulated, that is, some observed members would not have been observed if their 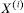 values had been otherwise, while some missing members would have been observed.

**Proposition 2**

The LMM/GLMM describes observed random subclusters of size *n* if (i) 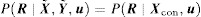, where 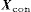 is a cluster-constant subvector of 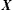; either (iia) 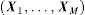 are exchangeable given *M* or (iib) 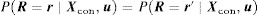 whenever 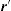 is a permutation of 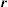; and (iii) 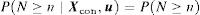.

Note that (iii) holds if the minimum possible observed cluster size is 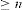, but is unlikely to hold otherwise; and if (iii) is replaced by the weaker condition 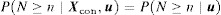, then 2011, 2007 and 2011 still hold, but 2009 may not.

### 5.4. Situations Where Complete- and Observed-Cluster Effects Differ

With the exceptions mentioned above (i.e., within-cluster fixed effects, and between-cluster and causal fixed effects in LMMs when 2007 holds), 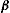 and 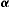 may not be so interpretable in terms of effects in observed clusters if 2007 or 2009 do not hold.

Suppose that 2009 with 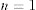 does not hold and 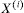 has a random effect. The between-observed-cluster effect of 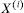 is given by 1995 with 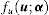 replaced by 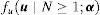. In particular, it does not reduce to 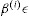 for the LMM unless 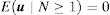. Similarly, the observed-cluster causal effect 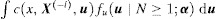 is, in general, not the same as the complete-cluster causal effect 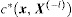; and the within-observed-cluster effect will not, in general, have mean 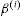 and variance implied by 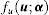.

In the following example, 2007 does not hold for 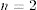. Suppose clusters are old people in a cohort study of cognitive function *Y*. A LMM is used, with a random effect for time because rate of cognitive decline varies between people. Assume a fixed effect for the intercept. The only missing data are due to death: 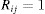 if person *i* is alive at time *j*; 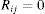 if dead. So, 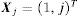, 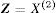, 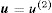 and missingness is monotone. Suppose people with more rapid decline (more negative 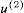) tend to die earlier. The within-complete-cluster effect of 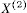 has mean 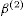 and variance 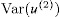. The mean and variance of the within-observed-cluster effect are functions of 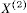: they both diminish as 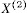 increases. This is because the subsample still alive at later times is enriched for high 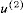. In this setting “complete-cluster” inference has been called inference for a hypothetical immortal cohort, and it has been suggested that “observed-cluster” inference (describing the population still alive at each timepoint) is of more interest (Dufouil et al., 2004). See Section 2011 and Web Appendix F for examples of between-cluster or causal effects differing in complete and observed clusters.

### 5.5. Observed Clusters Without Complete Clusters

Dunson et al. (2003), Chen et al. (2011) and Gueorguieva (2005) wanted observed-cluster inference when “complete clusters” do not exist, for example, toxicology experiments where clusters are litters. Dunson et al. and Gueorguieva assumed cluster-constant 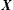, 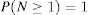 and 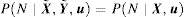. Chen et al. assumed 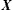 was cluster constant or a function of *j* (e.g., 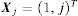), 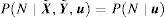 and 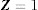. It can be seen that these methods give complete-cluster inference for a hypothetical population of complete clusters in which 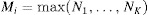 and from which the population of observed clusters would be generated by applying monotone missingness mechanism 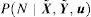. However, they do not *only* provide complete-cluster inference. When, as in Dunson et al. and Gueorguieva, 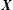 is cluster constant and 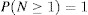, conditions (i), (iia) and (iii) of Proposition 2 hold with 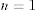, so 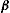 and 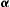 are also between-cluster or causal effects in observed clusters. When, as in Chen et al., 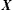 is cluster varying, 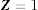 and 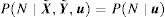, non-intercept elements of 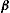 are within-observed-cluster effects.

## 6. Example: Psoriatic Arthritis

This example shows a model that ostensibly describes observed clusters but some of whose parameters relate only to a population of complete clusters with no obvious meaning. Husted et al. (2007) analyzed a cohort of 382 psoriatic arthritis (PsA) patients. Physical function was measured by the health assessment questionnaire score (HAQ). HAQ is semi-continuous: it is zero (no disability) with positive probability and otherwise varies continuously up to 3 (severe disability). 31% of the 2107 HAQ scores were zero. They separately modeled 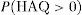 (the “binary-part”) and HAQ given 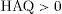 (the “continuous-part”), using, respectively, logistic regression with random intercept 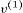 and linear regression with random intercept 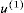. Both parts had the same covariates (sex, time since onset, etc.), and all covariates had fixed effects. Among the conclusions was that being female predicted higher HAQ when 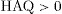, adjusting for other covariates.

Here, clusters are patients and “observed cluster” means a patient's set of non-zero scores. Su et al. (2009) noted that estimates for the continuous part might be biased because separate modeling of binary and continuous parts did not account for ICS caused by the model for the binary part determining the observed cluster size in the continuous part. So, they modified Husted et al.’s model by replacing 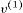 by 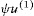, where 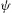 is unknown. They called this shared random-effect model the “latent-process model” (SAS code provided in Web Appendix G). They also used a correlated random effects model, but results were similar.

In the original (misspecified) model of Husted et al., the estimated sex effect in the continuous part was 0.181 (SE 0.051). In the latent-process model, it was 0.246 (SE 0.052) ( Table 1). We focus on the meaning of this latter estimate. We emphasize there is nothing intrinsically wrong with the latent-process model. It can validly be used to predict HAQ. What is important is not to misinterpret the parameters in the continuous part. As this is an LMM and sex is cluster-constant with fixed effect, the estimated sex effect, 0.246, describes the between-cluster effect in “complete clusters,” that is, in a hypothetical world in which all scores are somehow non-zero. The meaning and scientific interest of this hypothetical world, analogous to the world of “immortal cohorts,” is unclear.

**Table 1:** Estimates for latent process model and marginal model fitted to psoriatic arthritis data

	latent process model				marginal model	
	binary part		continuous part			
Parameter	estim	SE	estim	SE	estim	SE
Intercept	−0.9909	0.3556	0.1748	0.0555	0.263	0.0669
Age at onset	0.6392	0.1538	0.0984	0.0250	0.115	0.0267
Female	2.0037	0.3149	0.2461	0.0523	0.100	0.0580
PsA disease duration	0.0166	0.0220	0.0044	0.0032	0.004	0.0041
Actively inflamed joints	0.1380	0.0465	0.0243	0.0027	0.023	0.0045
Clinically deformed joints	0.0179	0.0238	0.0051	0.0031	0.007	0.0037
PASI score	0.1543	0.1017	0.0257	0.0134	−0.005	0.0237
Morning stiffness	1.5691	0.2018	0.1620	0.0262	0.273	0.0444
ESR	0.2971	0.1103	0.0374	0.0126	0.065	0.0232
Medication:						
NSAIDs	0.2960	0.2439	−0.0181	0.0280	−0.235	0.0467
DMARDs	0.3138	0.2197	0.0226	0.0272	0.003	0.0442
steroids	0.9927	0.4355	0.0481	0.0441	0.049	0.0553
Actively inflamed joints 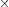 disease duration	0.0003	0.0031	−0.0005	0.0002	0.0000	0.0002
Clinically deformed joints 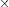 disease duration	0.0018	0.0011	0.0003	0.0001	0.0000	0.0001
Var(*u*)	4.2641	0.9001				
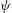			0.2074	0.0210		
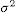			0.0779	0.0039		

Su et al. (2009) do not comment on the meaning of their estimated sex effect, but suppose one wished to interpret it as an effect in observed clusters, as done in Husted et al. (2007). As all the covariates have fixed effects, estimates for cluster-varying covariates can be interpreted unproblematically as within-cluster effects in complete or observed clusters. However, sex is cluster-constant. To illustrate the problem with interpreting the estimated sex effect, 0.246, as a between-cluster effect in observed clusters, we obtained the empirical Bayes estimate of each patient's random intercept 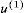. While the means of 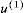 were 0.005 and 0.016 for men and women, respectively, means of 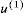 for observations on men and women when 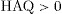 were 0.165 and 0.043. This difference arises because in the binary part of the model the estimated sex effect is 2.00 (SE 0.31), meaning that a woman was more likely to have 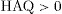 than a man with the same values of other covariates. So, if we compare a man and woman who both have 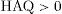 and have the same time since onset and other covariate values, we expect the woman's HAQ to be not 0.246 greater but only 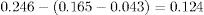 greater. Note that in Su et al.’s model, none of the conditions of Proposition 2 hold for any *n*.

We also used IEE to fit a model for 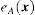, the conditional mean of HAQ given sex, time since onset, etc. and 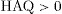 ( Table 1). The estimated sex effect is 0.100 (SE 0.031), which is close to the effect, 0.124, worked out above using empirical Bayes estimates.

In conclusion, the estimated sex effect in the continuous part of the latent-process model (and correlated random-effects model) describes the association between sex and HAQ in a hypothetical population of little scientific interest; for this dataset it overstates the size of the effect in the population of scientific interest. In further work, Su et al. (2011) found an association of genotype HLA-B27 with HAQ when 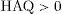. The same interpretation problem applies here: this association refers to the hypothetical “complete” clusters.

## 7. Discussion

We have shown that shared random-effect models do not always describe observed clusters, except for cluster-varying covariates with fixed effects or under the conditions of Proposition 2. The models of Dunson et al. (2003), Gueorguieva (2005) and Chen et al. (2011) are unnecessarily restrictive. They assume either cluster-constant 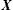 or that *N* does not depend on 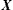. Proposition 2 shows 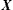 can be cluster varying if *N* depends only on cluster-constant elements. The assumptions required do, however, remain restrictive. WIEE relate to IPW for missing data. DWGEE2 give inference for a hypothetical population of complete clusters that is, in general, neither unique nor of scientific interest.

For binary *Y*, Li et al. (2011) used a correlated random-intercepts model with bridge distributions, so that 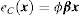. For a single binary *X*, they compared the log odds ratios in complete and observed clusters. They found the difference was small when the variance of the random intercepts or the correlation between them was small. However, when random-intercept variances and/or correlation are small, cluster size is only weakly informative; when size is strongly informative, inferences for complete and observed clusters will differ more. We replicated Li et al's study and found the two log odds ratios could differ by as much as 25% when 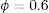, and 56% when 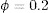 (see Web Appendix H).

We have assumed *Y* and 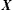 are observed in all members for which we wish to make inference. Dufouil et al. (2004) and Shardell and Miller (2008) give methods for when this is not so.

Having illustrated the danger of misinterpreting estimates, we recommend careful thought about which inference is of scientific interest and which analysis method will give it.

## 8. Supplementary Materials

Web Appendices referenced in Sections 3–7 are available with this paper at the Biometrics website on Wiley Online Library.

## References

[b1] Albert P, Follmann D, Fitzmaurice G, Davidian M, Verbeke G, Molenberghs G (2009). Shared-parameter models. Longitudinal Data Analysis.

[b2] Benhin E, Rao J, Scott A (2005). Mean estimating equation approach to analysing cluster-correlateddata with nonignorable cluster sizes. Biometrika.

[b3] Chen Z, Zhang B, Albert P (2011). A joint modeling approach to data with informative cluster size:Robustness to the cluster size model. Statistics in Medicine.

[b4] Dufouil C, Brayne C, Clayton D (2004). Analysis of longitudinal studies with death and drop-out: A casestudy. Statistics in Medicine.

[b5] Dunson D, Chen Z, Harry J (2003). A Bayesian approach for joint modeling of cluster size andsubunit-specific outcomes. Biometrics.

[b6] Gueorguieva R (2005). Comments about joint modelling of cluster size and binary andcontinuous subunit-specific outcomes. Biometrics.

[b7] Hoffman E, Sen P, Weinberg C (2001). Within-cluster resampling. Biometrika.

[b8] Huang Y, Leroux B (2011). Informative cluster size for subcluster-level covariates and weightedgeneralized estimating equations. Biometrics.

[b9] Husted J, Tom B, Farewell V, Schentag C, Gladman D (2007). A longitudinal study of the effect of disease activity and clinicaldamage on physical function over the course of psoriatic arthritis: Does theeffect change over time. Arthritis and Rheumatism.

[b10] Kurland B, Johnson L, Egleston B, Diehr P (2009). Longitudinal data with follow-up truncated by death: Match theanalysis method to research aims. Statistical Science.

[b11] Li X, Bandyopadhyay D, Lipsitz S, Sinha D (2011). Likelihood methods for binary responses of present components in acluster. Biometrics.

[b12] Neuhaus J, McCulloch C (2011). Estimation of covariate effects in generalized linear mixed modelswith informative cluster sizes. Biometrika.

[b13] Robins J-M, Rotnitzky A, Zhao L-P (1995). Analysis of semiparametric regression models for repeated outcomes inthe presence of missing data. Journal of the American Statistical Association.

[b14] Seaman S, Galati J, Jackson D, Carlin J (2013). What is meant by ‘missing at random’?. Statistical Science.

[b15] Shardell M, Miller R (2008). Weighted estimated equations for longitudinal studies with death andnon-monotone missing time-dependent covariates and outcomes. Statistics in Medicine.

[b16] Su L, Tom B, Farewell V (2009). Bias in 2-part mixed models for longitudinal semicontinuous data. Biostatistics.

[b17] Su L, Tom B, Farewell V (2011). A likelihood-based two-part marginal model for longitudinalsemi-continuous data. Statistical Methods in Medical Research.

[b18] Vansteelandt S (2007). On confounding, prediction and efficiency in the analysis oflongitudinal and cross-sectional clustered data. Scandinavian Journal of Statistics.

[b19] Wang M, Kong M, Datta S (2011). Inference for marginal linear models for clustered longitudinal datawith potentially informative cluster sizes. Statistical Methods in Medical Research.

[b20] Wang Z, Louis T (2003). Matching conditional and marginal shapes in binary random interceptmodels with a bridge distribution function. Biometrika.

[b21] Williamson J, Datta S, Satten G (2003). Marginal analyses of clustered data when cluster size is informative. Biometrics.

