# Supplementary material for: Methods for Observed-Cluster Inference When Cluster Size Is Informative: A Review and Clarifications
Source: Biometrics. 2014 Jan 30;70(2):449–56. doi: 10.1111/biom.12151 (PMC4312901; doi:10.1111/biom.12151)
Supplement: Supplementary file 1 — Supporting Information. [file biom0070-0449-sd1.pdf]

Web-based Supplementary Materials for "Methods for Observed-Cluster Inference when Cluster Size is Informative: a Review and Clarifications' by Shaun Seaman, Menelaos Pavlou and Andrew Copas

Web Appendix A: Proof of Proposition 1

First, suppose that data are MCAR and equation (1) holds. MCAR means that membership of the observed cluster cannot depend on the $\boldsymbol{X}$ and Y values in the complete cluster. Therefore $E(Y_H \mid \boldsymbol{X}_H = \boldsymbol{x}, N, M)$ is equal to a (possibly) weighted average of $E(Y_1 \mid \boldsymbol{X}_1 = \boldsymbol{x}, M), \ldots, E(Y_M \mid \boldsymbol{X}_M = \boldsymbol{x}, M)$ with weights that may depend on N and $\boldsymbol{x}$. If (1) holds, this weighted average is equal to $E(Y_1 \mid \boldsymbol{X}_1 = \boldsymbol{x})$ no matter what the weights are. Hence, $E(Y_H \mid \boldsymbol{X}_H = \boldsymbol{x}, N)$ does not depend on N. Second, suppose that data are MCAR and MWEP and $N \perp\!\!\!\perp M$. MWEP means that each member of the complete cluster is equally likely to be included in the observed cluster. Therefore the relation between Y and $\boldsymbol{X}$ in a randomly chosen member of the observed cluster will be the same as the relation in a randomly chosen member of the corresponding complete cluster. Since MCAR and $N \perp\!\!\!\perp M$ guarantees that the latter relation does not depend on N, nor does $E(Y_H \mid \boldsymbol{X}_H, N)$ depend on N.

Web Appendix B: Examples of MAR and MNAR mechanisms generating ICS and NICS

There are no covariates in any of the following examples.

Example 1: MNAR and NICS

Suppose Y is binary and $P(R_j = 0 \mid Y_j = 0) = 1\ \forall j$. Then $e_T = e_A = 1$, the data are MNAR and cluster size is non-informative.

Example 2: MAR and NICS

Suppose $M = 4$ for all clusters and $\tilde{\boldsymbol{Y}} = (1,0,0,1)^T$ or $\tilde{\boldsymbol{Y}} = (0,1,0,1)^T$ with equal probability. Suppose that $P\{\boldsymbol{R} = (1,1,1,1)^T \mid (Y_1, Y_2) = (1,0)\} = P\{\boldsymbol{R} = (1,1,0,0)^T \mid (Y_1, Y_2) = (0,1)\} = 1$. Then $E(Y_H \mid N = 2) = E(Y_H \mid N = 4) = 0.5$, and so $e_T = e_A = 0.5$.

Example 3: MNAR and ICS

Suppose $M_i = 3$ for all clusters, $u_1, \ldots, u_K \sim N(0,1)$, $Y_{ij} \mid u_i \sim N(u_i, 1)$, $P(N = 0) = 0$ and logit $P(N_i = k \mid \tilde{\boldsymbol{Y}}_i, u_i, N_i \geqslant k)\} = u_i - 1$ $(k = 1,2)$. Then $e_T = 0$ and calculations show that $e_A = -0.20$.

Example 4: MAR and ICS

Suppose $M_i = 3$ for all clusters, $u_1, \ldots, u_K \sim N(0,1)$, $Y_{ij} \mid u_i \sim N(u_i, 1)$, $P(N_i = 0) = 0$ and logit $P(N_i = k \mid \tilde{\boldsymbol{Y}}_i, u_i, N_i \geqslant k)\} = Y_{ik} - 1$ $(k = 1,2)$. Calculations show that $e_T = 0.12$ and $e_A = -0.18$.

Web Appendix C: Proof that weighted GEE with weights M/N consistently estimate $\boldsymbol{\beta}$ in the model $e_C(\boldsymbol{x}) = g^{-1}(\boldsymbol{\beta}^T\boldsymbol{x})$ when data are MWEP and $P(N \geqslant 1) = 1$

If data on complete clusters were available, ordinary (unweighted) 'complete-data' GEE could be used to estimate $\boldsymbol{\beta}$. These GEE are $\sum_{i=1}^K \boldsymbol{U}(\boldsymbol{\beta}; \tilde{\boldsymbol{X}}_i, \tilde{\boldsymbol{Y}}_i) = \boldsymbol{0}$, where $\boldsymbol{U}(\boldsymbol{\beta}; \tilde{\boldsymbol{X}}, \tilde{\boldsymbol{Y}}) = \boldsymbol{D}(\tilde{\boldsymbol{X}}, \boldsymbol{\beta})^T \boldsymbol{V}(\tilde{\boldsymbol{X}})\{\tilde{\boldsymbol{Y}} - \tilde{\boldsymbol{\mu}}(\tilde{\boldsymbol{X}}, \boldsymbol{\beta})\}$, with $\tilde{\boldsymbol{\mu}}(\tilde{\boldsymbol{X}}, \boldsymbol{\beta}) = (g^{-1}(\boldsymbol{X}_1^T\boldsymbol{\beta}) \ldots, g^{-1}(\boldsymbol{X}_M^T\boldsymbol{\beta}))$, $\boldsymbol{D}(\tilde{\boldsymbol{X}}, \boldsymbol{\beta}) = \partial\tilde{\boldsymbol{\mu}}(\tilde{\boldsymbol{X}}, \boldsymbol{\beta})/\partial\boldsymbol{\beta}$ and $\boldsymbol{V}(\tilde{\boldsymbol{X}})$ being the working covariance matrix. The true value of $\boldsymbol{\beta}$ obeys $E\{\boldsymbol{U}(\boldsymbol{\beta}; \tilde{\boldsymbol{X}}, \tilde{\boldsymbol{Y}})\} = \boldsymbol{0}$ and hence the solution $\hat{\boldsymbol{\beta}}$ to these GEE consistently estimates $\boldsymbol{\beta}$ (Tsiatis AA, 2006, Semiparametric Theory and Missing Data, Springer, New York, pp 29–31).

The weighted GEE that use only observed members and weight them by M/N are

$$\sum_{i=1}^{K} \boldsymbol{D}(\tilde{\boldsymbol{X}}_i, \boldsymbol{\beta})^T \boldsymbol{V}(\tilde{\boldsymbol{X}}_i) \boldsymbol{W}_{\text{ep}}(\boldsymbol{R}_i)\{\tilde{\boldsymbol{Y}}_i - \tilde{\boldsymbol{\mu}}(\tilde{\boldsymbol{X}}_i, \boldsymbol{\beta})\} \tag{14}$$

where $\boldsymbol{W}_{\text{ep}}(\boldsymbol{R}_i) = \text{diag}(R_1, \ldots, R_M)M/N$.

When $P(R_j \mid \tilde{\boldsymbol{X}}, \tilde{\boldsymbol{Y}}, N) = N/M \; \forall j$ (i.e. data are MWEP) and $P(N \geqslant 1) = 1$,

$$
\begin{aligned}
& E\left[\boldsymbol{D}(\tilde{\boldsymbol{X}}, \boldsymbol{\beta})^T \boldsymbol{V}(\tilde{\boldsymbol{X}}) \boldsymbol{W}_{\text{ep}}(\boldsymbol{R})\{\tilde{\boldsymbol{Y}} - \tilde{\boldsymbol{\mu}}(\tilde{\boldsymbol{X}}, \boldsymbol{\beta})\}\right] \\
&= E_{\tilde{\boldsymbol{X}}, \tilde{\boldsymbol{Y}}, N} E_{\boldsymbol{R} \mid \tilde{\boldsymbol{X}}, \tilde{\boldsymbol{Y}}, N}\left[\boldsymbol{D}(\tilde{\boldsymbol{X}}, \boldsymbol{\beta})^T \boldsymbol{V}(\tilde{\boldsymbol{X}}) \boldsymbol{W}_{\text{ep}}(\boldsymbol{R})\{\tilde{\boldsymbol{Y}} - \tilde{\boldsymbol{\mu}}(\tilde{\boldsymbol{X}}, \boldsymbol{\beta})\}\right] \\
&= E_{\tilde{\boldsymbol{X}}, \tilde{\boldsymbol{Y}}, N} E_{\boldsymbol{R} \mid N, M}\left[\boldsymbol{D}(\tilde{\boldsymbol{X}}, \boldsymbol{\beta})^T \boldsymbol{V}(\tilde{\boldsymbol{X}}) \boldsymbol{W}_{\text{ep}}(\boldsymbol{R})\{\tilde{\boldsymbol{Y}} - \tilde{\boldsymbol{\mu}}(\tilde{\boldsymbol{X}}, \boldsymbol{\beta})\}\right] \\
&= E_{\tilde{\boldsymbol{X}}, \tilde{\boldsymbol{Y}}, N}\left(\boldsymbol{D}(\tilde{\boldsymbol{X}}, \boldsymbol{\beta})^T \boldsymbol{V}(\tilde{\boldsymbol{X}}) \; E_{\boldsymbol{R} \mid N, M}\{\boldsymbol{W}_{\text{ep}}(\boldsymbol{R})\} \; \{\tilde{\boldsymbol{Y}} - \tilde{\boldsymbol{\mu}}(\tilde{\boldsymbol{X}}, \boldsymbol{\beta})\}\right) \\
&= E_{\tilde{\boldsymbol{X}}, \tilde{\boldsymbol{Y}}, N}\left(\boldsymbol{D}(\tilde{\boldsymbol{X}}, \boldsymbol{\beta})^T \boldsymbol{V}(\tilde{\boldsymbol{X}})\{\tilde{\boldsymbol{Y}} - \tilde{\boldsymbol{\mu}}(\tilde{\boldsymbol{X}}, \boldsymbol{\beta})\}\right) \\
&= E\{\boldsymbol{U}(\boldsymbol{\beta}; \tilde{\boldsymbol{X}}, \tilde{\boldsymbol{Y}})\}
\end{aligned}
$$

Hence the solution to these weighted GEE consistently estimates $\boldsymbol{\beta}$.

Web Appendix D: DWGEE2

Huang and Leroux's (2011) method is slightly more general than we described in the article, in that they proposed that one could weight for only a categorical subvector $\boldsymbol{X}^{\text{sub}}$ of $\boldsymbol{X}$ and adjust for the rest of $\boldsymbol{X}$ via a regression model. In this Appendix we treat this general method of Huang and Leroux (2011).

Let $\boldsymbol{X}^{\text{sub}}$ be either $\boldsymbol{X}$ or a subvector of $\boldsymbol{X}$, and be such that $\boldsymbol{X}^{\text{sub}}$ is categorical. Denote the possible values of $\boldsymbol{X}^{\text{sub}}$ by $\boldsymbol{a}_1, \ldots, \boldsymbol{a}_L$. Suppose that each observed cluster is a subcluster of a corresponding complete cluster, with every complete cluster containing at least one member with $\boldsymbol{X}^{\text{sub}} = \boldsymbol{a}_l$ for each $l = 1, \ldots, L$. For each complete cluster, let B_l denote a random variable with $P(B_l = j \mid \tilde{\boldsymbol{X}}, \tilde{\boldsymbol{Y}}, M) = I(\boldsymbol{X}_j^{\text{sub}} = \boldsymbol{a}_l)/M^{(l)}$, where $M^{(l)} = \sum_j I(\boldsymbol{X}_j^{\text{sub}} = \boldsymbol{a}_l)$ is the number of members with $\boldsymbol{X}^{\text{sub}} = \boldsymbol{a}_l$ in the complete cluster. So, B_l denotes the index of a randomly selected member with $\boldsymbol{X}^{\text{sub}} = \boldsymbol{a}_l$ from the complete cluster.

Let $N^{(l)}$ denote the number of members in the observed cluster that have $\boldsymbol{X}^{\text{sub}} = \boldsymbol{a}_l$, and

let J_l denote a random variable with $P(J_l = j \mid \tilde{\boldsymbol{X}}, \tilde{\boldsymbol{Y}}, M) = R_j I(\boldsymbol{X}_j^{\text{sub}} = \boldsymbol{a}_l)/N^{(l)}$. So, J_l denotes the index of a randomly selected member with $\boldsymbol{X}^{\text{sub}} = \boldsymbol{a}_l$ from the observed cluster. Suppose that the model $E(Y_{B_l} \mid \boldsymbol{X}_{B_l} = \boldsymbol{x}) = g^{-1}(\boldsymbol{\beta}^T\boldsymbol{x})$, for the conditional expectation of Y given $\boldsymbol{X}$ in the population formed by drawing from each complete cluster at random one member with each value of $\boldsymbol{X}^{\text{sub}}$, is correctly specified and the true value of $\boldsymbol{\beta}$ is $\boldsymbol{\beta}_0$. Let

$$\boldsymbol{U}(Y, \boldsymbol{X}; \beta) = \frac{\partial \mu(\boldsymbol{X}, \boldsymbol{\beta})}{\partial \boldsymbol{\beta}} V(\boldsymbol{X})\{Y - \mu(\boldsymbol{X}, \boldsymbol{\beta})\}$$

where $\mu(\boldsymbol{X}, \boldsymbol{\beta}) = g^{-1}(\boldsymbol{X}^T\boldsymbol{\beta})$ and $V(\boldsymbol{X})$ is a working variance of Y given $\boldsymbol{X}$. [Note that $\boldsymbol{U}$ and V are not the same as the $\boldsymbol{U}$ and $\boldsymbol{V}$ used in Appendix C: the notation is being recycled.] Then $\boldsymbol{\beta}_0$ is the solution to

$$E\left\{\sum_{l=1}^{L} \boldsymbol{U}(Y_{B_l}, \boldsymbol{X}_{B_l}; \boldsymbol{\beta}_0)\right\} = E\left\{\sum_{j=1}^{M} \frac{\boldsymbol{U}(Y_j, \boldsymbol{X}_j; \boldsymbol{\beta}_0)}{M^{(\boldsymbol{X}_j^{\text{sub}})}}\right\} = \boldsymbol{0} \tag{15}$$

and would be consistently estimated by the GEE

$$\sum_{i=1}^{K}\sum_{j=1}^{M_i} \frac{\boldsymbol{U}(Y_{ij}, \boldsymbol{X}_{ij}; \boldsymbol{\beta})}{M_i^{(\boldsymbol{X}_j^{\text{sub}})}} = \boldsymbol{0}.$$

Suppose we have a model for $E(N^{(l)} \mid \boldsymbol{W})$, where $\boldsymbol{W}$ is a vector of cluster-level covariates, and let $\pi_l(\boldsymbol{W})$ denote the estimated value of $E(N^{(l)} \mid \boldsymbol{W})$ from this model. Let Y_j^*, $\boldsymbol{X}_j^*$ and $\boldsymbol{X}_j^{\text{sub}*}$ denote Y, $\boldsymbol{X}$ and $\boldsymbol{X}^{\text{sub}}$ for the jth member of the observed cluster ($j = 1, \ldots, N$). The DWGEE2 estimating equations are

$$\sum_{i=1}^{K}\sum_{j=1}^{N_i} \frac{\boldsymbol{U}(Y_{ij}^*, \boldsymbol{X}_{ij}^*; \boldsymbol{\beta})}{\pi_{\boldsymbol{X}_{ij}^{\text{sub}*}}(\boldsymbol{W}_i)}$$

These can be written equivalently as

$$\sum_{i=1}^{K}\sum_{j=1}^{M_i} R_{ij} \frac{\boldsymbol{U}(Y_{ij}, \boldsymbol{X}_{ij}; \boldsymbol{\beta})}{\pi_{\boldsymbol{X}_{ij}^{\text{sub}}}(\boldsymbol{W}_i)}$$

Now make the following three assumptions. First,

$$\pi_l(\boldsymbol{W}) \to \pi_{0l}(\boldsymbol{W}) \text{ as } K \to \infty \qquad \forall l \tag{16}$$

where $\pi_{0l}(\boldsymbol{W})$ denotes the true value of $E(N^{(l)} \mid \boldsymbol{W})$. Second,

$$\exists \delta > 0 \text{ such that } P\{\pi_{0l}(\boldsymbol{W}) > \delta\} = 1 \qquad \forall l \tag{17}$$

Third,

$$E\{\boldsymbol{U}(Y_{J_l},\boldsymbol{X}_{J_l};\boldsymbol{\beta}_0)\mid N^{(l)}=n,\boldsymbol{W}\}=E\{\boldsymbol{U}(Y_{J_l},\boldsymbol{X}_{J_l};\boldsymbol{\beta}_0)\mid N^{(l)}>0,\boldsymbol{W}\}\quad\forall l,\ \text{and}\ \forall n>0 \tag{18}$$

Note that in the special case where $\boldsymbol{X}^{\text{sub}}=\boldsymbol{X}$, a sufficient condition for equation (18) to be true is $E(Y_{J_l}\mid N^{(l)}=n,\boldsymbol{W})=E(Y_{J_l}\mid N^{(l)}>0,\boldsymbol{W})$ $\forall l$ and $\forall n>0$.

Proposition 1A

When expressions (16), (17) and (18) are true,

$$E\left\{\sum_{j=1}^{N}\frac{\boldsymbol{U}(Y_j^*,\boldsymbol{X}_j^*;\boldsymbol{\beta}_0)}{\pi_{0\boldsymbol{X}_j^{\text{sub}*}}(\boldsymbol{W})}\right\}=\sum_{l=1}^{L}E_W[E\{\boldsymbol{U}(Y_{J_l},\boldsymbol{X}_{J_l};\boldsymbol{\beta}_0)\mid\boldsymbol{W},N^{(l)}>0\}]. \tag{19}$$

Proof of Proposition 1A

Let $\tilde{\boldsymbol{Y}}^*=(Y_1^*,\ldots,Y_N^*)^T$ and $\tilde{\boldsymbol{X}}^*=(\boldsymbol{X}_1^*,\ldots,\boldsymbol{X}_N^*)$. Then

$$\begin{aligned}
&E\left\{\sum_{j=1}^{N}\frac{\boldsymbol{U}(Y_j^*,\boldsymbol{X}_j^*;\boldsymbol{\beta}_0)}{\pi_{0\boldsymbol{X}_{ij}^{\text{sub}*}}(\boldsymbol{W})}\right\}\\
&=\sum_{l=1}^{L}E\left\{\sum_{j=1}^{N}I(\boldsymbol{X}_j^{\text{sub}*}=\boldsymbol{a}_l)\frac{\boldsymbol{U}(Y_j^*,\boldsymbol{X}_j^*;\boldsymbol{\beta}_0)}{\pi_{0l}(\boldsymbol{W})}\right\}\\
&=\sum_{l=1}^{L}E_WE_{N^{(l)}|\boldsymbol{W}}E_{\tilde{\boldsymbol{X}}^*,\tilde{\boldsymbol{Y}}^*,N|N^{(l)},W}\left\{\sum_{j=1}^{N}\frac{I(\boldsymbol{X}_j^{\text{sub}*}=\boldsymbol{a}_l)}{E(N^{(l)}\mid\boldsymbol{W})}\boldsymbol{U}(Y_j^*,\boldsymbol{X}_j^*;\boldsymbol{\beta}_0)\right\}\\
&=\sum_{l=1}^{L}\boldsymbol{h}_l
\end{aligned}$$

where

$$\begin{aligned}
\boldsymbol{h}_l &= E_WE_{N^{(l)}|\boldsymbol{W}}E_{\tilde{\boldsymbol{X}}^*,\tilde{\boldsymbol{Y}}^*,N|N^{(l)},W}\left\{\sum_{j=1}^{N}\frac{I(\boldsymbol{X}_j^{\text{sub}*}=\boldsymbol{a}_l)}{E(N^{(l)}\mid\boldsymbol{W})}\boldsymbol{U}(Y_j^*,\boldsymbol{X}_j^*;\boldsymbol{\beta}_0)\right\} \quad (20)\\
&= E_W\left[\frac{1}{E(N^{(l)}\mid\boldsymbol{W})}E_{N^{(l)}|\boldsymbol{W}}E_{\tilde{\boldsymbol{X}}^*,\tilde{\boldsymbol{Y}}^*,N|N^{(l)},W}\left\{\sum_{j=1}^{N}I(\boldsymbol{X}_j^{\text{sub}*}=\boldsymbol{a}_l))\,\boldsymbol{U}(Y_j^*,\boldsymbol{X}_j^*;\boldsymbol{\beta}_0)\right\}\right]\\
&= E_W\left[\frac{P(N^{(l)}>0\mid\boldsymbol{W})}{E(N^{(l)}\mid\boldsymbol{W})}E_{N^{(l)}|\boldsymbol{W},N^{(l)}>0}E_{\tilde{\boldsymbol{X}}^*,\tilde{\boldsymbol{Y}}^*,N|N^{(l)},W}\left\{\sum_{j=1}^{N}I(\boldsymbol{X}_j^{\text{sub}*}=\boldsymbol{a}_l)\,\boldsymbol{U}(Y_j^*,\boldsymbol{X}_j^*;\boldsymbol{\beta}_0)\right\}\right]\\
&= E_W\left[\frac{P(N^{(l)}>0\mid\boldsymbol{W})}{E(N^{(l)}\mid\boldsymbol{W})}E_{N^{(l)}|\boldsymbol{W},N^{(l)}>0}\left\{N^{(l)}\;E[\boldsymbol{U}(Y_{J_l},\boldsymbol{X}_{J_l};\boldsymbol{\beta}_0)\mid N^{(l)},\boldsymbol{W}]\right\}\right]
\end{aligned}$$

Assuming equation (18) is true, this becomes

$$\begin{aligned}
\boldsymbol{h}_l &= E_W\left[\frac{P(N^{(l)}>0 \mid \boldsymbol{W})}{E(N^{(l)} \mid \boldsymbol{W})}E_{N^{(l)}\mid \boldsymbol{W},N^{(l)}>0}\left\{N^{(l)}\ E[\boldsymbol{U}(Y_{J_l},\boldsymbol{X}_{J_l};\boldsymbol{\beta}_0)\mid N^{(l)}>0,\boldsymbol{W}]\right\}\right] \\
&= E_W\left\{\frac{P(N^{(l)}>0 \mid \boldsymbol{W})}{E(N^{(l)} \mid \boldsymbol{W})}E[\boldsymbol{U}(Y_{J_l},\boldsymbol{X}_{J_l};\boldsymbol{\beta}_0)\mid N^{(l)}>0,\boldsymbol{W}]\ E(N^{(l)}\mid \boldsymbol{W},N^{(l)}>0)\right\} \\
&= E_W\left\{\frac{E(N^{(l)} \mid \boldsymbol{W})}{E(N^{(l)} \mid \boldsymbol{W})}E[\boldsymbol{U}(Y_{J_l},\boldsymbol{X}_{J_l};\boldsymbol{\beta}_0)\mid N^{(l)}>0,\boldsymbol{W}]\right\} \qquad (21) \\
&= E_W\left\{E[\boldsymbol{U}(Y_{J_l},\boldsymbol{X}_{J_l};\boldsymbol{\beta}_0)\mid N^{(l)}>0,\boldsymbol{W}]\right\}
\end{aligned}$$

as required. Therefore Proposition 1A is proven.

Now also make the assumption that

$$E\left\{\boldsymbol{U}(Y_{B_l},\boldsymbol{X}_{B_l};\boldsymbol{\beta})\mid \boldsymbol{W}\right\} = E\left\{\boldsymbol{U}(Y_{J_l},\boldsymbol{X}_{J_l};\boldsymbol{\beta})\mid \boldsymbol{W},N^{(l)}>0\right\} \qquad \forall l=1,\ldots,L \qquad (22)$$

This is a missing-data assumption that makes inference for $\boldsymbol{\beta}$ possible using just the data on observed clusters. Essentially, it means that the distribution of Y given $\boldsymbol{X}$ is the same in members with $\boldsymbol{X}^{\text{sub}} = a_l$ randomly chosen from complete clusters as it is in members with $\boldsymbol{X}^{\text{sub}} = a_l$ randomly chosen from those observed clusters that have at least one member with $\boldsymbol{X}^{\text{sub}} = a_l$. Note that in the special case where $\boldsymbol{X}^{\text{sub}} = \boldsymbol{X}$, a sufficient condition for equation (22) to be true is $E(Y_{B_l}\mid \boldsymbol{W}) = E(Y_{J_l}\mid \boldsymbol{W},N^{(l)}>0)\ \forall l$.

Proposition 2A

When expressions (16), (17), (18) and (22) are true, the solution to the DWGEE2 converges to $\boldsymbol{\beta}_0$ as $K\to\infty$.

Proof of Proposition 2A

From Proposition 1A and equation (22), we have

$$\begin{aligned}
E\left\{\sum_{j=1}^{N}\frac{\boldsymbol{U}(Y_j^*,\boldsymbol{X}_j^*;\boldsymbol{\beta}_0)}{\pi_{0\boldsymbol{X}_{ij}^{\text{sub}*}}(\boldsymbol{W})}\right\} &= \sum_{l=1}^{L}E_W E\left\{\boldsymbol{U}(Y_{B_l},\boldsymbol{X}_{B_l};\boldsymbol{\beta}_0)\mid \boldsymbol{W}\right\} \\
=\sum_{l=1}^{L}E\left\{\boldsymbol{U}(Y_{B_l},\boldsymbol{X}_{B_l};\boldsymbol{\beta}_0\right\}
\end{aligned}$$

So, it follows from equation (15) that the solution to the DWGEE2 converges to $\boldsymbol{\beta}_0$ as $K \to \infty$. Therefore Proposition 2A is proven.

When observed clusters are complete in themselves and do not arise from complete clusters as a result of missing data, equations (15) and (22) and Proposition 2A are statements about a population of complete clusters that does not exist. Proposition 1A, on the other hand, reveals what the estimand of DWGEE2 is without making reference to a population of complete clusters. There is more than one way to interpret Proposition 1A. One way is that it shows that DWGEE2 are making inference for a population of members formed in the following way. For each $l = 1, \ldots, L$, each cluster in the population of observed clusters contributes a member with $\boldsymbol{X}^{\text{sub}} = \boldsymbol{a}_l$. If $N^{(l)} > 1$, i.e. if an observed cluster contains more than one member with $\boldsymbol{X}^{\text{sub}} = \boldsymbol{a}_l$, one of these $N^{(l)}$ members is chosen at random. If $N^{(l)} = 0$, i.e. an observed cluster contains no members with $\boldsymbol{X}^{\text{sub}} = \boldsymbol{a}_l$, then another observed cluster with the same value of $\boldsymbol{W}$ but with $N^{(l)} > 0$ is selected at random and a member with $\boldsymbol{X}^{\text{sub}} = \boldsymbol{a}_l$ chosen at random for this cluster instead. This is like hot-deck imputation. A second way of interpreting Proposition 2A is that it shows that DWGEE2 are making inference for a population of members formed in the following way. Values of $\boldsymbol{W}$ are sampled at random from the distribution of $\boldsymbol{W}$ (i.e. the distribution of $\boldsymbol{W}$ in the population of clusters). For each sampled value of $\boldsymbol{W}$ and for each $l = 1, \ldots, L$, a cluster with that value of $\boldsymbol{W}$ and with $N^{(l)} > 0$ is chosen at random and an observed member with $\boldsymbol{X}^{\text{sub}} = \boldsymbol{a}_l$ chosen at random from this cluster. These two populations of members are equivalent.

The problem with DWGEE2 when observed clusters are complete in themselves rather than being subclusters of complete clusters is that there is no obvious reason to make one choice of which covariates to include in $\boldsymbol{W}$ rather than another choice. Suppose, for example, that clusters are patients. If $\boldsymbol{W}$ includes sex, then the borrowing of an observation from a patient with $N^{(l)} > 0$ to 'impute' a 'missing' observation in another patient with $N^{(l)} = 0$ will be

done only from a patient of the same sex, whereas if $\boldsymbol{W}$ does not include sex, then a patient of either sex will be used. Should sex be included or not, and why? The same question could be asked of age or any number of other cluster-constant covariates.

Web Appendix E: Proof of Proposition 2

Conditions i) and either iia) or iib) ensure that each observed cluster can be considered to be the result of a MWEP mechanism applied to a random permutation of its corresponding complete cluster, and thus that, provided $P(N \geqslant n) = 1$, $\tilde{\boldsymbol{X}}_{(H_n)}$ is independent of $\boldsymbol{u}$. In the case where $P(N \geqslant n) < 1$, the additional condition iii) ensures that the distribution of $\boldsymbol{u}$ in observed clusters of size n does not depend on the value of any cluster-constant covariates and is equal to $f_u(\boldsymbol{u}; \boldsymbol{\alpha})$.

Web Appendix F: Examples where complete- and observed-cluster effects differ

Example 5 below illustrates that between-cluster effects in observed clusters and complete clusters can differ. Example 6 illustrates the difference for causal effects. Both examples concern LMMs, and the difference arises in each example because $\boldsymbol{u} \perp\!\!\!\perp \tilde{\boldsymbol{X}}_{(H_n)} \mid N \geqslant n$ does not hold for $n = 1$.

EXAMPLE 5: Clusters are patients. Their symptom severity Y is to be measured once before and once after treatment ($M = 2$). To allow variability in Y to be greater after treatment, a random treatment effect is included. The intercept has a fixed effect. So, $\boldsymbol{X}_{i1} = (1, 0)^T$ and $\boldsymbol{X}_{i2} = (1, 1)^T$; $\boldsymbol{Z}_{i1} = \boldsymbol{Z}_{i2} = 1$; and $\boldsymbol{u} = u^{(1)}$. Suppose that in patients with $u^{(1)} \geqslant 0$ only the before-treatment Y is observed (i.e. $\boldsymbol{R}_i = (1, 0)^T$); in patients with $u^{(1)} < 0$ only the after-treatment measure is observed (i.e. $\boldsymbol{R} = (0, 1)^T$). So, $N = 1$ for all clusters, and patients whose Y is observed when untreated (respectively, treated) have positive (respectively,

negative) $u^{(1)}$. The between-complete-cluster effect of treatment is $\beta^{(2)}$, but the between-observed-cluster effect is $\beta^{(2)} + E\{u^{(1)} \mid \boldsymbol{R} = (0,1)^T\} - E\{u^{(1)} \mid \boldsymbol{R} = (1,0)^T\} < \beta^{(2)}$. Here the complete-cluster effect is probably of more interest.

Example 6: Clusters are patients. Their symptom severity Y is to be measured $M = 8$ times. Let $R_{ij} = 1$ if patient i has symptoms at time j; $R_{ij} = 0$ if no symptoms. So, 'observed-cluster inference' means conditional on having symptoms. Patients are randomly allocated treatment or no treatment independently at each time. Treatment does not affect whether or not symptoms are present, but does affect their severity if they are present. Interest is in the causal effect of treatment on severity when patients have symptoms. A random treatment effect is included. The intercept and time variables have fixed effects. So, $\boldsymbol{X}_{ij} = (1, X^{(2)}_{ij}, j-1)^T$, where $X^{(2)}$ is treatment; $P(X^{(2)}_{ij} = 1) = 0.5$; $\boldsymbol{Z}_{ij} = X^{(2)}_{ij}$; and $\boldsymbol{u} = u^{(2)}$. Suppose that patients with positive treatment effect ($u^{(2)} > 0$) always have symptoms at times 1–4 but not at times 5–8 ($\boldsymbol{R}_i = (1,1,1,1,0,0,0,0)^T$), whereas all patients with negative or no effect ($u^{(2)} \leqslant 0$) have symptoms at times 5–8 but not at 1–4 ($\boldsymbol{R}_i = (0,0,0,0,1,1,1,1)^T$). In complete clusters the mean causal treatment effect adjusted for time is $\beta^{(2)} = 0$; in observed clusters it is positive at times 1–4 and negative at 5–8.

Web Appendix G: SAS code for fitting latent-process model of Su et al. (2009)

The following SAS code (except for minor modifications to make it produce empirical Bayes estimates of random effects) was kindly made available by Li Su.

```
LIBNAME haqdirect "<name of directory where data is>";


* Suppose hazdata.sas7bdat is name of datafile and the variable names are
PTNO, HAQ, AGEPSA2, SEX, ARTHDUR, ACTIVEJT, DEFORMED, PASI2, STIFFAM, ESR2,
```

MED1, MED2, MED3, ART_ACT and ART_DEF.

PTNO is patient number and HAQ is the outcome variable;

```
DATA twopm;
set haqdirect.haqdata;
keep PTNO HAQ AGEPSA2 SEX ARTHDUR ACTIVEJT DEFORMED PASI2 STIFFAM ESR2 MED1
MED2 MED3 ART_ACT ART_DEF;
run;

ods rtf file="<name of file to put results in>"
startpage=NO;

title "<title for analysis in output file>";
PROC NLMIXED DATA=haqdirect.haqdata ABSFCONV=0.001 ABSCONV=0.000 GCONV=0
ABSGCONV=0 COV;
PARMS gamma0=-0.9909 gamma1=0.639 gamma2=2.0036 gamma3=0.0165 gamma4=0.1379
gamma5=0.0177 gamma6=0.1545 gamma7=1.5693 gamma8=0.2971 gamma9_1=0.296
gamma9_2=0.3137 gamma9_3=0.9928 gamma10=0.0003 gamma11=0.0018
beta0=0.1747 beta1=0.0983 beta2=0.2461 beta3=0.0044 beta4=0.0248
beta5=0.0056 beta6=0.0256 beta7=0.1621 beta8=0.0374 beta9_1=-0.0181
beta9_2=0.0226 beta9_3=0.0480 beta10=-0.0005 beta11=0.0003  sig=0.0783
logsigeps2= 1.45 alpha=0.2094; * initial values;
BOUNDS sig > 0;
sigeps2=exp(logsigeps2);
MU=beta0 + beta1*AGEPSA2 + beta2*SEX +  beta3*ARTHDUR + beta4*ACTIVEJT +
```

```
beta5*DEFORMED+ beta6*PASI2 + beta7*STIFFAM + beta8*ESR2+ beta9_1*MED1 +
beta9_2*MED2 + beta9_3*MED3 + beta10*ART_ACT + beta11*ART_DEF +alpha*EPS;
* IDENTITY LINK FOR LME MODEL;
LOGITP=gamma0 + gamma1*AGEPSA2 + gamma2*SEX +  gamma3*ARTHDUR +
gamma4*ACTIVEJT + gamma5*DEFORMED+ gamma6*PASI2 + gamma7*STIFFAM +
gamma8*ESR2+ gamma9_1*MED1 + gamma9_2*MED2 + gamma9_3*MED3 +
gamma10*ART_ACT + gamma11*ART_DEF +EPS;
* LOGIT LINK FOR  LOGISTIC  MODEL;
P=1/(1+EXP(-LOGITP)); * PROBABILITY OF NON-ZERO HAQ;
LOGLIKE=(1-HAQBIN)*LOG(1-P)+(HAQBIN)*(LOG(P) -
0.5*((HAQ-MU)/sqrt(SIG))**2 - LOG(SQRT(sig*8*ATAN(1))));
MODEL HAQ ~ GENERAL(LOGLIKE);
RANDOM EPS  ~ NORMAL(0,sigeps2) SUBJECT=PTNO;
PREDICT EPS out=EPS;
ESTIMATE "sigeps2" exp(logsigeps2);
RUN;


ods rtf close;
```

Web Appendix H: Replicating Li et al.'s (2011) Study

In Section 2 of their article, Li et al. (2011) describe a study of the difference between $e_c(\boldsymbol{x})$ and $e_A(\boldsymbol{x})$. They assumed that $M_i = 16$ for all clusters, X is a cluster-constant covariate, logit $P(R_{ij} = 1 \mid X_i) = \beta_0 + \beta_1 X_i + v_i$ and logit $P(Y_{ij} = 1 \mid X_i) = \alpha_0 + \alpha_1 X_i + u_i$, where $\beta_0 = \alpha_0 = 1$, $\beta_1 = \alpha_1 = -1$, and u and v are random effects whose marginal distributions are bridge distributions with rescaling parameter ϕ and whose correlation is approximately

equal to ρ (see Li et al., 2011, for full details). The variance of a bridge distribution with rescaling parameter ϕ is $\pi^2(\phi^{-2}-1)/3$ (Wang and Louis, 2003). Li et al. considered scenarios where $\phi = 0.4$, 0.6 or 0.8, and $\rho = 0.4$, 0.6 or 0.8.

The use of the bridge distribution means that logit $e_C(x) = \text{logit } P(Y = 1 \mid X = x) = \phi\alpha_0 + \phi\alpha_1 x$ (Wang and Louis, 2003), and hence $\log \text{OR}_\text{C} = \text{logit } e_C(1) - \text{logit } e_C(0)$, the marginal log odds ratio comparing $X = 1$ with $X = 0$ in the population of complete clusters, equals $\phi\alpha_1$. Li et al. (2011) used simulation to calculate logit $e_A(x) = \text{logit } P(Y = 1 \mid X = x, R = 1)$ and hence $\log \text{OR}_\text{A} = \text{logit } e_A(1) - \text{logit } e_A(0)$, the marginal log odds ratio in the population of observed clusters.

We replicated Li et al.'s study and also extended it to consider scenarios where $\beta_1 = \alpha_0 = 1$ and $\beta_0 = \alpha_1 = -1$. To calculate $e_A(x)$ we simulated 1,000,000 clusters for each combination of ϕ, ρ and x values ($x = 0$ and $x = 1$). We also calculated $e_C(x)$ from these simulated clusters and verified that logit $e_C(x)$ was equal to its theoretical value $\phi\alpha_0 + \phi\alpha_1 x$.

Web Table 1 shows the log odds ratios $\log \text{OR}_\text{C}$ and $\log \text{OR}_\text{A}$ in complete and observed clusters, respectively, when $\beta_0 = 1$ and $\beta_1 = -1$. Also shown, in the final column of the table, is the percentage difference between $\log \text{OR}_\text{C}$ and $\log \text{OR}_\text{A}$, i.e. $100\% \times \{\log \text{OR}_\text{C} - \log \text{OR}_\text{A}\}/\log \text{OR}_\text{A}$. Web Table 2 shows the corresponding results when $\beta_0 = -1$ and $\beta_1 = 1$.

The percentage difference between the two log odds ratios increases as ϕ (and hence the variance of u and v) and ρ (and hence the correlation between u and v) increase. When $\beta_0 = 1$ and $\beta_1 = -1$ (Web Table 1), $\log \text{OR}_\text{C}$ overstates $\log \text{OR}_\text{A}$; when $\beta_0 = -1$ and $\beta_1 = 1$ (Web Table 2), it understates it.

ϕ	ρ	log OR_C	log OR_A	% difference
0.2	0.4	-0.200	-0.158	26.8
0.2	0.6	-0.200	-0.142	41.2
0.2	0.8	-0.200	-0.128	56.1
0.4	0.4	-0.400	-0.334	19.6
0.4	0.6	-0.400	-0.308	29.7
0.4	0.8	-0.400	-0.285	40.2
0.6	0.4	-0.600	-0.531	12.9
0.6	0.6	-0.600	-0.504	19.1
0.6	0.8	-0.600	-0.480	25.0
0.8	0.4	-0.800	-0.753	6.2
0.8	0.6	-0.800	-0.734	9.1
0.8	0.8	-0.800	-0.717	11.6

Web Table 1: marginal log odds ratios for complete and observed clusters, and percentage difference ($\beta_0 = 1$ and $\beta_1 = -1$).

ϕ	ρ	log OR_C	log OR_A	% difference
0.2	0.4	-0.200	-0.266	-24.8
0.2	0.6	-0.200	-0.330	-39.4
0.2	0.8	-0.200	-0.464	-56.9
0.4	0.4	-0.400	-0.516	-22.5
0.4	0.6	-0.400	-0.613	-34.7
0.4	0.8	-0.400	-0.771	-48.1
0.6	0.4	-0.600	-0.725	-17.2
0.6	0.6	-0.600	-0.810	-25.9
0.6	0.8	-0.600	-0.919	-34.7
0.8	0.4	-0.800	-0.883	-9.4
0.8	0.6	-0.800	-0.929	-13.9
0.8	0.8	-0.800	-0.979	-18.2

Web Table 2: marginal log odds ratios for complete and observed clusters, and percentage difference ($\beta_0 = -1$ and $\beta_1 = 1$).
